# Which preventive measures might protect health care workers from SARS?

**DOI:** 10.1186/1471-2458-9-81

**Published:** 2009-03-13

**Authors:** Wei-Qing Chen, Wen-Hua Ling, Ci-Yong Lu, Yuan-Tao Hao, Zhong-Ning Lin, Li Ling, Jian Huang, Gang Li, Guang-Mei Yan

**Affiliations:** 1 Department of Biostatistics and Epidemiology, School of Public Health, Sun Yat-Sen University, 74 Zhongshan Road II, Guangzhou, PR China; 2 Department of Nutrition, School of Public Health, Sun Yat-Sen University, 74 Zhongshan Road II, Guangzhou, PR China; 3 Department of Preventive Medicine, School of Public Health, Sun Yat-Sen University, 74 Zhongshan Road II, Guangzhou, PR China; 4 Department of Surgery, the Second Affiliated Hospital, Sun Yat-Sen University, 107 Yanjiangxi Road, Guangzhou, PR China; 5 Department of Infection, the Third Affiliated Hospital, Sun Yat-Sen University, Dinggang, Shipai, Guangzhou, PR China; 6 Department of Pharmacology, Zhongshan School of Medicine, Sun Yat-Sen University, 74 Zhongshan Road II, Guangzhou, PR China

## Abstract

**Background:**

Despite the use of a series of preventive measures, a high incidence of severe acute respiratory syndrome (SARS) was observed among health care workers (HCWs) during the SARS epidemic. This study aimed to determine which preventive measures may have been effective in protecting HCWs from infection, and which were not effective.

**Methods:**

A retrospective study was performed among 758 'frontline' health care workers who cared for SARS patients at the Second Affiliated Hospital and the Third Affiliated Hospital of Sun Yat-sen University. The HCWs with IgG against SARS and those without IgG against SARS were respectively defined as the "case group" and the "control group", and logistic regression was conducted to explore the risk factors for SARS infection in HCWs.

**Results:**

After adjusting for age, gender, marital status, educational level, professional title, and the department in which an individual worked, the results of a multivariate logistic regression analysis indicated that incidence of SARS among HCWs was significantly and positively associated with: performing tracheal intubations for SARS patients, methods used for air ventilation in wards, avoiding face-to-face interaction with SARS patients, the number of pairs of gloves worn by HCWs, and caring for serious SARS cases.

**Conclusion:**

Some measures, particularly good air ventilation in SARS wards, may be effective in minimizing or preventing SARS transmission among HCWs in hospitals.

## Background

Severe acute respiratory syndrome (SARS), a viral respiratory illness caused by the coronavirus SARS-CoV, was possibly the first globally significant occupational disease to emerge in the 21st century, making healthcare work potentially hazardous [1]. This was indicated by the high incidence of SARS observed among health care workers (HCWs) in the epidemic of SARS, especially during its earlier stages [2-5]. In China, from a total of 5323 SARS cases, 966 (over 18%) were HCWs, and in the early period of the SARS epidemic, near 90% of the SARS patients were frontline HCWs [6]. In Hong Kong, a total of 384 (22.1%) of 1739 suspected or confirmed SARS patients were hospital workers [7]. Generally, SARS outbreaks first originated in hospitals where SARS patients were treated and subsequently spread to communities from there [8,9].

Several studies indicated that HCWs coming into direct or indirect contact with SARS patients in wards had a greatly increased risk of contracting SARS-Cov, despite some strict infection control measures being taken [7,10,11]. A similar situation also arose in the Second Affiliated Hospital and the Third Affiliated Hospital of Sun Yat-sen University during the epidemic of SARS in 2003. A total of 846 HCWs worked on the frontline of caring for SARS patients in the two affiliated hospitals and 112 of them contracted SARS during this time. Throughout the whole period of the SARS epidemic, a series of infection control and protective measures were employed in the two affiliated hospitals. But, why were some of HCWs infected by SARS, and some of them were not? The objective of this study was to determine which preventive measures used were effective in protecting HCWs from SARS, and which were not effective. To answer this question, we conducted a retrospective study of HCWs who worked at the frontline in the two affiliated hospitals during the SARS epidemic.

## Methods

### Study population

In mid-May 2003, about 4 months after the initial SARS outbreak in Guangzhou, a retrospective study was conducted in HCWs working at the frontline of the SARS epidemic, providing primary care in the Second Affiliated Hospital and the Third Affiliated Hospital of Sun Yat-sen University, where the first and second outbreak of SARS among HCWs occurred in the early stage of SARS epidemic in Guangzhou. Among a total of 846 frontline HCWs who tended to SARS patients from the two hospitals, 758 (89.2%) who were on duty during the investigation were surveyed, and they included HCWs from all departments involved in the care of SARS patients in the two hospitals. But, those who were off-duty during the survey were excluded. During the SARS epidemic, a total of 112 HCWs working on the frontline were diagnosed suffering from "SARS" according to a case definition of SARS by the Ministry of Health, China [12], and 90 of them were successfully interviewed, giving a response rate of 80.4% (90/112). Written informed consent was obtained from all the participants prior to enrollment after a detailed explanation of the study objectives and requirements of the survey. The Ethical Committee of the Sun Yat-sen University approved the study.

### Definition of a SARS case

A SARS case was defined using the criteria for probable SARS cases provided by the Health Ministry of China [12]. Criteria for probable and suspected SARS cases included travel to a SARS epidemic area in the 2 weeks before the onset of symptoms or close contact with a probable SARS patient; fever of ≥ 38°C; chest x-ray abnormalities; normal or decreased leukocycte count; and no response to treatment by antimicrobial drugs.

In the present study, 10 mL of peripheral venous blood was collected from all the subjects, and the serum was separated and stored at -70°C. Immunoglobulin (Ig) G against SARS-CoV was detected using an enzyme-linked immunosorbent assay (ELISA) [13]. Among the 758 surveyed HCWs, 91 ones (80 cases were diagnosed suffering from "SARS" and 11 ones were not diagnosed suffering from "SARS") had IgG antibodies against SARS, and the prevalence rate of IgG antibodies against SARS was 12.01% for the total samples [13]. Furthermore, the prevalence of IgG antibodies against SARS-CoV was 88.9% (80/90) for HCW with SARS [86.3% (63/73) in the Second Affiliated Hospital and 100.0% (17/17) in the Third Affiliated Hospital], and 1.6% (11/668) for HCW without SARS who worked on frontline of SARS [2.8% (8/288) in the Second Affiliated Hospital and 0.8% (3/380) in the Third Affiliated Hospital] [13].

### Data collection

A standardized interview with a structured questionnaire was used to obtain the following information in mid-May 2003, about 4 months after the initial SARS outbreak in Guangzhou. (1) Socio-demographic characteristics, including: age, gender, marital status, educational level, professional title, and in which department did you work? (2) History of SARS patient care, including: i) Did you receive any special training for how to handle SARS? ii) Did you ever perform a tracheotomy? iii) Did you ever perform tracheal intubations? iv) Did you ever care for "Super Spreading SARS cases"? (3) Use of personal protective and control measures, including: i) How many gowns did you wear while you cared for SARS patients? ii) How many multilayered cotton masks did you wear while you cared for SARS patients? iii) How many pairs of gloves did you wear while you cared for SARS patients? iv) With what frequency did you wear shoe covers while you cared for SARS patients? vi) With what frequency did you wear a cap while you cared for SARS patients? (vii) With what frequency did you wear a face shield while you worked in SARS wards? i) With what frequency did you wear goggles while you performed operations on SARS patients? (4) Health-related behaviors included: i) With what frequency did you wash uncovered skin after you cared for SARS patients? ii) With what frequency did you wash hands after you cared for SARS patients? iii) With what frequency did you wash your nasal cavity after you cared for SARS patients? iv) With what frequency did you wash your mouth after you cared for SARS patients? (5) Other relevant control measures were: i) What type of air ventilation system was used in your office and in SARS wards? ii) What type of hand-washing equipment was used in your office? More details about the name, definition and value of these variables are listed in Table 1.

**Table 1:** Variables and definition

**Variable name**	**Definition**
*Socio-demographics*	
Age	Year
Gender	1 = Male, 2 = Female
Marital status	1 = Unmarried, 2 = Married, 3 = Others
Educational level	1 = Senior school, 2 = Technical secondary school, 3 = Junior college, 4 = university, 5 = Master degree, 6 = PhD
Professional title	1 = Doctor, 2 = Nurse, 3 = Health attendant, 4 = Technician in laboratory, 5 = Others
Department	1 = SARS ward, 2 = Emergency department/Fever clinic, 3 = Infectious disease, 4 = Respiratory disease, 5 = Others
*Use of personal protective and control measures*	
Number of gowns worn	0 = Single, 1 = Double
Number of multilayered cotton mask worn	0 = Single, 1 = Double
Number of pairs of gloves worn	0 = Single, 1 = double
Frequency of wearing shoe cover	0 = Never, 1 = Sometimes, 2 = Often, 3 = Every time
Frequency of wearing cap	0 = Never, 1 = Sometimes, 2 = Often, 3 = Every time
Frequency of face shield in SARS ward	0 = Never, 1 = Sometimes, 2 = Often, 3 = Every time
Frequency of wearing goggles while performing operation for SARS patients	0 = Never, 1 = Sometimes, 2 = Often, 3 = Every time
*Health-related behaviors*	
Frequency of washing uncovered skin after caring for SARS patients	0 = Never, 1 = Sometimes, 2 = Often, 3 = Every time
Frequency of washing hands after caring for SARS patients	0 = Never, 1 = Sometimes, 2 = Often, 3 = Every time
Frequency of washing nasal cavity after caring for SARS patients	0 = Never, 1 = Sometimes, 2 = Often, 3 = Every time
Frequency of washing oral cavity after caring for SARS patients	0 = Never, 1 = Sometimes, 2 = Often, 3 = Every time
*SARS patient care*	
Special training for SARS	0 = No, 1 = Yes
Performing tracheotomy	0 = No, 1 = Yes
Performing tracheal intubations	0 = No, 1 = Yes
Caring for "Super Spreading Patient"	0 = No, 1 = Yes
Avoiding face to face while caring for patient	0 = Never, 1 = Sometimes, 2 = Often, 3 = Every time
*Other relevant control measures*	
Method of air ventilation in offices and SARS wards	1 = Artificial central ventilation (windows were closed in wards), 2 = Natural ventilation (windows were opened in wards), 3 = Natural ventilation and additional electronic exhaust fan (windows were opened in wards, at the same time, electronic exhaust fans were used for improving air circulation in wards)
Type of equipment for washing hands	1 = Automatic tap, 2 = Non-automatic tap, 3 = Others

### Data analysis

HCWs who had IgG against SARS (91 cases = 80 cases with SARS and 11 cases without SARS) and those without both IgG against SARS and SARS (657 cases) were defined as the "case group" and "control group", respectively. 10 HCWs had been previously diagnosed as SARS, but their IgG against SARS test was negative, so that they were excluded from the data analysis. Logistic regression was conducted to explore the risk factors for SARS infection among HCWs and odd ratios (ORs) and 95% confidence intervals were used to assess the association of SARS infection with the factors studied. Univariate analysis was performed at first for each risk factor. Factors with P < 0.1 were included in a multivariate logistic regression analysis and analyzed using a forward-stepwise procedure. In the multivariate logistic regression analysis, age, gender, marital status, educational level, professional title, and the department in which the HCW worked were controlled as potential confounding factors. The entry and exit criteria were set at *P *= 0.05 and *P *= 0.10, respectively. List-wise deletion was used in the multivariate analyses. All the *P *values were two-tailed, and a *P *< 0.05 value was considered statistically significant, unless otherwise mentioned.

All the statistical analyses were performed using SPSS 11.0 for Windows [14].

## Results

### Socio-demographic characteristic of the surveyed subjects

Table 2 presents general information about the surveyed subjects provided by the two affiliated hospitals.

**Table 2:** Socio-demographic characteristics of HCWs in the two affiliated hospitals

	**Second Affiliated Hospital**		**Third Affiliated Hospital**	
		
**Socio-demographic characteristics**	**No.**	**%**	**No.**	**%**
***Age (years)***				
<26	152	42.1	113	28.5
26~30	83	23.0	126	31.7
31~35	59	16.3	67	16.9
36~40	37	10.3	39	9.8
>40	30	8.3	52	13.1
***Gender***				
Male	80	22.2	101	25.5
Female	281	77.8	295	74.5
***Marital status***				
Unmarried	209	57.9	172	43.3
Married	147	40.7	213	53.7
Others	5	1.4	12	3.0
***Educational level***				
Senior school	17	4.7	46	11.6
Technical secondary school	157	43.5	138	34.8
Junior college	58	16.1	72	18.1
University	74	20.5	43	10.8
MD/PhD	55	15.2	98	24.7
***Department***				
SARS ward	227	68.2	111	33.8
Emergency department/Fever clinic	25	7.5	52	15.9
Infectious disease department	5	1.5	120	36.6
Respiratory diseases department	42	12.6	15	4.6
Others	34	10.2	30	9.1
***Professional title***				
Doctor	105	29.1	134	33.8
Nurse	199	55.1	174	43.8
Health attendant	15	4.2	40	10.1
Technician in laboratory	25	6.9	13	3.3
Others	17	4.7	36	9.1

### Logistic regression analysis

Table 3 presents the results of univariate logistic regression analysis. Among the eighteen surveyed risk factors, fifteen factors were significantly associated with SARS infection in HCWs, with the exceptions being "Frequency of wearing face shield in SARS ward", "Frequency of washing hands after caring for SARS patients", and "Frequency of washing nasal cavity after caring for SARS patients". See Table 3.

**Table 3:** Univariate logistic regression analysis

**Risk factors**		**SARS case**	**Control**	**OR (95% CI)**	**P value**
***Number of gowns worn***	Single	42	189	2.12 (1.36~3.31)	<0.001
	Double	49	468		
***Number of multilayered cotton mask***	Single	32	116	2.53 (1.57~4.07)	<0.001
***Worn***	Double	59	541		
***Number of pairs of gloves worn***	Single	81	400	5.20 (2.65~10.23)	<0.001
	Double	10	257		
***Frequency of wearing shoe cover***	Never	55	237	3.80 (2.24~6.45)	<0.001
	Sometimes	8	26	5.04 (2.04~12.48)	<0.001
	Often	7	50	2.29 (0.93~5.67)	>0.05
	Every time	21	344	1.00	
***Frequency of wearing cap***	Never	68	380	1.79 (1.03~3.10)	<0.05
	Sometimes	3	63	0.48 (0.14~1.67)	>0.05
	Often	2	34	0.59 (0.13~2.65)	>0.05
	Every time	18	180	1.00	
***Frequency of wearing face shield in ***	Never	89	506	4.05 (0.54~30.34)	>0.05
***SARS ward***	Sometimes	1	107	0.22 (0.01~3.56)	>0.05
	Often	0	21	---	---
	Every time	1	23	1.00	
***Frequency of wearing goggles while ***	Never	88	483	7.83 (1.07~57.63)	<0.05
***performing operation for SARS patients***	Sometimes	2	103	0.84 (0.07~9.45)	>0.05
	Often	0	28	---	---
	Every time	1	43	1.00	
***Frequency of washing uncovered skin***	Never	62	339	3.29 (1.29~8.43)	<0.05
***after caring for SARS patients***	Sometimes	17	142	2.16 (0.77~6.05)	>0.05
	Often	7	86	1.47 (0.45~4.79)	>0.05
	Every time	5	90	1.00	
***Frequency of washing hands after caring***	Never	23	186	0.89 (0.52~1.51)	>0.05
***for SARS patients***	Sometimes	5	35	1.03 (0.38~2.75)	>0.05
	Often	18	113	1.14 (0.64~2.06)	>0.05
	Every time	45	323	1.00	
***Frequency of washing nasal cavity after***	Never	66	398	3.21 (0.98~10.53)	>0.05
***caring for SARS patients***	Sometimes	20	154	2.51 (0.72~8.77)	>0.05
	Often	2	47	0.82 (0.13~5.13)	>0.05
	Every time	3	58	1.00	
***Frequency of washing oral cavity after***	Never	69	376	3.26 (1.15~9.21)	<0.05
***caring for SARS patients***	Sometimes	17	147	2.05 (0.67~6.33)	>0.05
	Often	1	63	0.28 (0.03~2.59)	>0.05
	Every time	4	71	1.00	
***Special training for SARS***	No	74	421	2.44 (1.41~4.23)	<0.001
	Yes	17	236		
***Performing tracheotomy***	Yes	6	11	4.15 (1.50~11.50)	<0.01
	No	85	646		
***Performing tracheal intubations***	Yes	16	17	8.03 (3.90~16.56)	<0.001
	No	75	640		
***Caring for "Super Spreading Patient "***	Yes	69	268	4.55 (2.75~7.54)	<0.001
	No	22	389		
***Avoiding face to face while caring for ***	Never	40	182	1.00	
***patient***	Sometimes	23	200	0.64 (0.36~1.10)	>0.05
	Often	24	173	0.53 (0.31~0.93)	<0.05
	Every time	4	113	0.16 (0.06~0.46)	<0.001
***Method of air ventilation in offices and ***	ACV	20	295	1.00	
***SARS wards***	NV	54	333	0.28 (0.14~0.54)	<0.001
	NVEEF	17	29	0.17 (0.06~0.25)	<0.001
***Type of equipment for washing hands***	AT	5	125	1.00	
	NAT	85	509	4.18 (1.66~10.51)	<0.001
	Others	1	23	1.09 (0.12~9.74)	>0.05

ACV: Artificial central ventilation; NV: Natural ventilation; NVEEF: Natural ventilation and additional electronic exhaust fan; AT: Automatic tap; NAT: Non-automatic tap

After adjusting for age, gender, marital status, educational level, professional title, and the department in which the individual worked, a multivariate logistic regression model identified five variables associated with altered risk of contracting SARS at a significance level of 0.05 (Table 4). They were: performing tracheal intubations for SARS patients, insufficient methods used for air ventilation in wards, avoiding face-to-face interaction while caring for SARS patients, the number of pairs of gloves worn by the HCW, and caring for "Super Spreading SARS Cases". The result of the Hosmer-Lemeshow goodness of fit for the model was: χ^2 ^= 4.739, df = 7, and P > 0.05.

**Table 4:** Multivariate logistic regression analysis

**Risk factors**	**OR (95% CI)**	**P value**
***Caring for "Super Spreading Patient" (No = 0: yes = 1)***	3.57(1.94~6.57)	<0.001
***Avoiding face to face contact while caring for SARS patients***		
Never	1.00	
Sometimes	0.67(0.36~1.24)	>0.05
Often	0.30(0.10~0.90)	<0.05
Every time	0.30(0.15~0.60)	<0.001
***Number of pairs of gloves worn (Double = 0: single = 1)***	4.13(1.99~8.55)	<0.001
***Method of air ventilation in office and SARS ward***		
Artificial central ventilation	1.00	
Natural ventilation	0.40(0.18~0.88)	<0.05
Natural ventilation and additional electronic exhaust fan	0.27(0.16~0.63)	<0.01
***Performing tracheal intubations (No = 0: yes = 1)***	2.76(1.16~6.53)	<0.05

Performing tracheal intubation for SARS patients and caring for "Super Spreading SARS Cases" significantly increased the risk of SARS infection among HCWs working on the frontline. In contrast, wearing multiple (2) pairs of gloves could protect HCWs from SARS infection. Compared with wards with artificial central ventilation, those with natural ventilation or with both natural ventilation and electronic exhaust fans at the same time significantly decreased the probability of HCWs being infected with SARS-CoV. A much lower incidence rate of SARS was found among HCWs who either usually or consistently avoided face-to-face contact with SARS patients in their care.

## Discussion

It was hypothesized that performing certain high-risk procedures, such as nasopharyngeal aspiration, bronchoscopy, endotracheal intubation, airway suction, and cardiopulmonary resuscitation, might increase the rate of SARS-Cov shedding occurring in a SARS patient's respiratory secretions, thereby increasing the risk to HCWs of contracting SARS while performing such procedures [15]. The results of the present study demonstrate that performing tracheal intubations was highly associated with incidence of SARS among HCWs. Therefore, the results imply that adequate personal protective equipment is required when conducting certain high-risk procedures which may contribute to the presence of infectious droplets in the environment.

It was hypothesized that the primary mode of SARS transmission was via droplets spread through close person-to-person contact [1], and this was strongly supported by the occurrence of clusters of cases among HCWs caring for SARS patients and family members of SARS patients [16,17]. In the present study, we found that avoiding face-to-face contact with SARS patients while caring for them could significantly reduce the probability of HCWs contracting the virus. This may be due to decreased exposure to infected droplets resulting from this practice. This result implies that HCWs could use appropriate personal protective measures (such as avoiding face-to-face contact with SARS patients) to protect themselves from SARS infection while they are caring for patients with SARS.

There is some evidence that longer range airborne transmission may have played a role in the spread of the SARS virus in some settings, such as in the outbreak of SARS in wards with faulty ventilation in the Prince of Wales Hospital of Hong Kong [17], in the transmission of SARS on an aircraft [18], and in the community outbreak at Amoy Gardens in Hong Kong [19]. The results of the present study also indicate that airborne transmission might have been a contributing factor in spread of SARS in 2003. Compared with ventilation through artificial central air-conditioners in the wards, natural ventilation alone and natural ventilation enhanced by an additional electronic exhaust fan at the same time could significantly reduce the risk of HCWs contracting SARS in the wards. In wards with artificial central ventilation, windows were closed which might lead to much lower air flow and much higher viral load in the wards, and HCWs were easily infected with the SARS virus while working in such an environment. By contrast, the windows of wards with natural ventilation and natural ventilation enhanced by an additional electronic exhaust fans were opened, and the air flow and the exchange rate of air in the wards were high, which might greatly decrease the density of the SARS virus in the wards and may also reduce the probability of HCWs contracting the virus.

SARS-Cov may be shed from a SARS patient's respiratory secretion and feces, and the latter may further contaminate objects in the ward. The protective gown, gloves, multilayered cotton mask, and head and foot coverings wore by HCWs may also be contaminated while caring for SARS patients. It has been shown that SARS-Cov may remain viable for considerable periods on a dry surface (up to 24 hours) [16] and is stable in feces and urine at room temperature for at least 1 to 2 days and 4 days in stool from patients with diarrhea [19]. Hence, touching surfaces or objects that are contaminated with SARS-CoV may introduce the virus into the mucous membranes of the eye, nose, or possibly the mouth. It is believed that nominally 'clean' areas may be contaminated if an HCW wears a piece of protective clothing contaminated with SARS patients' secretions into the area. For this reason, HCWs must wear two layers of gown, gloves, multilayered cotton mask, head and foot covering in SARS wards and discard the outer layer before entering clean areas, in order to prevent fomite transmission to other areas [20]. This study proved that wearing two layers of gloves significantly protected HCWs from SARS compared with wearing a single layer of gloves, but we did not find that wearing double layers of gowns, multilayered cotton masks, and head and foot coverings were associated with HCWs being protected from SARS. This might be due to the fact that almost of all the procedures involved in caring for patients were done with the hands; hence gloves were more highly contaminated by SARS patients' secretions.

A small number of severely infected patients or super-spreading patients appeared to play a disproportionate role in the spread of the disease to HCWs. For instance, several clusters of SARS outbreaks in hospitals can be traced to such patients in Hong Kong, Singapore, and Toronto [2,4,5]. It had been hypothesized that these patients might have a relatively depressed immune system with associated high viral loads and may be unduly facilitating transmission of the virus. In the present study, the same index patient led to the two clusters of SARS outbreaks among HCWs in the two affiliated hospitals. Statistical analysis showed that caring for a "Super-spreading Patient" significantly increased the risk of HCWs suffering from SARS. In light of this, a series of stringent infection control measures should be required when HCWs care for patients suspected of being SARS super-spreaders.

Several limitations of the study ought to be mentioned here. First, our investigation was limited to two affiliated hospitals of Sun Yat-sen University. This is not representative of all of the hospitals in which patients with SARS were admitted and cared for in Guangzhou. Therefore, this is a typical case investigation. Second, ventilation in the wards was not objectively assessed for some reason, meaning that we could not exactly evaluate the influence of the ventilation in the wards on the transmission of SARS among HCWs. Third, we could not trace the tree structure of the primary, secondary, and third class cases, which prevented us from clarifying the association of the HCWs infected by SARS with the index case directly or indirectly. Fourth, some factors, such as oxygen therapy and bi-level positive airway pressure ventilation were found to be related to nosocomial infection of SARS in other study [21], were not included in the present study, which indicated that we missing an opportunity to find some effective measures for protecting HCWs from SARS or to assess their effect. Fifth, in the early stage of SARS epidemic, the diagnosis of SARS was based on the history of epidemiology, signs and symptoms suggested by the Health Ministry of China [12], not on the directive biomarkers of SARS-CoV or antibodies against SARS-CoV, which might lead to over reporting "SARS" cases or missing identifying inapparent infection or subclinical infection. This might be the reason that 80 of 90 HCWs with "SARS" and 11 of 668 subjects without "SARS" were seropositive. Sixth, some prevention measures were usually employed at the same time in SARS wards, which meant that these measures were highly correlated. In this situation, multivariate statistical analysis might omit some effective measures in the final model due to multicollinearity. Seventh, 10.8% of frontline HCWs who cared for SRAS patients were not included in the present study, which was the reason that the number of HCWs involved in intubation in the present study was less than our previous study [13], which might cause to underestimate the association of the intubation with the nosocomial infection of SARS. Finally, although we identified several preventive measures which were effective for protecting HCWs from SARS, we could not eliminate the inefficiencies of other adopted measures, due to the fact that we utilized a retrospective rather than an interventional study design.

## Conclusion

In summary, good air ventilation in wards and a series of simple control and preventive measures might decrease or prevent SARS transmission among HCWs in hospitals.

## Competing interests

The authors declare that they have no competing interests.

## Authors' contributions

W-QC contributed to study design, data analysis and paper writing. WHL participated in study design and administered the study. CYL, YTH, ZLL, LL were responsible for the field investigation and data collection. JH and GL organized the field investigation. GMY participated in study design and administered the study. All authors read and approved the final manuscript.

## Pre-publication history

The pre-publication history for this paper can be accessed here:


